# Impact of Gout on Left Atrial Function: A Prospective Speckle-Tracking Echocardiographic Study

**DOI:** 10.1371/journal.pone.0108357

**Published:** 2014-09-24

**Authors:** Kuo-Li Pan, Jing-Chi Lin, Chun-Liang Lin, Mien-Cheng Chen, Shih-Tai Chang, Chang-Min Chung, Jen-Te Hsu

**Affiliations:** 1 Division of Cardiology, Chang Gung Memorial Hospital, Chiayi, Taiwan; 2 Division of Allergy and Immunology and Rheumatology, Chang Gung Memorial Hospital, Chiayi, Taiwan; 3 Division of Nephrology, Chang Gung Memorial Hospital, Chiayi, Taiwan; 4 The Graduate Institute of Clinical Medical Sciences, Chang Gung University, Taoyuan, Taiwan; 5 Division of Cardiology, Kaohsiung Chang Gung Memorial Hospital, Kaohsiung, Taiwan; University of Bologna, Italy

## Abstract

The purpose of our study was to evaluate the left ventricular (LV) and left atrial (LA) function in patients with gout. A total of 173 patients underwent a comprehensive Doppler-echocardiography examination. Participants were divided into four groups–Stage 0: control (n = 35), Stage I: asymptomatic hyperuricemia (n = 30), Stage II: gouty arthritis without tophi (n = 58), and Stage III: tophaceous gout (n = 50). Serum uric acid levels were not significantly different between stage I, II and III. Stage III patients demonstrated a higher ratio of the transmitral and myocardial peak early diastolic velocities (E/Em) (10.50±3.18 vs. 8.58±2.07; P = 0.008), and larger maximal LA volume index (LAVi) (29.60±9.89 vs. 20.07±4.76 ml/m^2^; P<0.001) compared with controls. Stage III patients had decreased LV global longitudinal systolic strain (LVε) compared with controls (−20.2±3.06 vs. −21.79±2.27; P = 0.002). Stage III patients also had decreased peak atrial longitudinal strain rate during ventricular systole (ALSR_syst_), peak atrial longitudinal strain rate during ventricular early diastole (ALSR_early_), and peak atrial longitudinal strain rate during ventricular late diastole (ALSR_late_) compared with controls (1.73±0.48 vs. 2.05±0.55 1/s, −1.44±0.53 vs. −2.07±0.84 1/s, −2.07±0.7 vs. −2.66±0.91 1/s, respectively; all P<0.005). Multiple regression analysis revealed severity of gout had an independent negative impact on LA pump function (ALSR_late_). In conclusion, gout caused LV diastolic dysfunction, LV subclinical systolic dysfunction and LA reservoir, conduit, and booster pump dysfunction.

## Introduction

Gout is a common metabolic disorder characterized by hyperuricemia and chronic inflammation [1]. The severity of gout can be divided into four stages, including asymptomatic hyperurecemia, acute gout attack, intercritical gout, and chronic tophaceous gout. Numerous studies have reported elevated serum uric acid (UA) as a risk factor of coronary heart disease [2], atrial fibrillation [3], cardiac mortality [4], and is associated with poor outcomes in patients with heart failure [5]. Elevated UA is also associated with left ventricular (LV) hypertrophy in patients without underlying cardiovascular disease [6], and is also associated with diastolic dysfunction in patients with heart failure [7]. Hyperuricemia accelerates the occurrence cardiovascular disease due to LV remodeling.

Clinically, severity of gout, rather than hyperuricemia, is more representative of the chronic inflammation in patients with gout. Epidemiological studies have found gout is associated with increased incidence of coronary heart disease and stroke. However, the exact pathological mechanism underlying this association is unclear [8]. It remains unclear whether hyperuricemia is the sole contributor to organic heart remodeling in patients with gout.

LA remodeling has been previously proposed to be a predictor of cardiovascular disease, including atrial fibrillation, congestive heart failure, and cardiovascular death [9]–[11]. There is a paucity of data regarding the association between LA function and severity of gout. The aim of the current study was to investigate the gout impairs LV and LA function or not.

## Methods

### Subjects and Study Design

Between October 2010 and February 2013 patients diagnosed with gout including asymptomatic hyperuricemia, gouty arthritis without tophi, and tophaceous gout were enrolled in this prospective study. Age-matched individuals without hyperuricemia or gout served as controls. Exclusion criteria included the presence of moderate or severe valvular heart disease, dilated or hypertrophic cardiomyopathy, LV systolic dysfunction (defined as an LV ejection fraction <50%), cardiac arrhythmia, pacemaker implantation, congenital heart disease, coronary artery disease and a history of cardiac surgery. Patients were also excluded if they had poor echocardiographic images for analysis.

All study protocols were approved by the Chang Gung Medical Foundation Institutional Review Board (IRB100-3022B) in our hospital and signed informed consent was obtained from all patients. Background and demographic data was collected from enrolled patients and included age, sex, height, body weight, systolic blood pressure, serum creatinine level, UA level, and absence or presence of hypertension, diabetes mellitus (DM), hypercholesterolemia, and medications for gouty arthritis. Asymptomatic hyperuricemia was defined as serum UA ≥7 mg/dl without a history of clinically painful joints histories. The diagnosis of gouty arthritis was made according to the American College of Rheumatology (ACR)/Wallace criteria [12]. Patients who had been diagnosed with gouty arthritis using ACR criteria and received medication for gouty arthritis were also included in the gouty arthritis group. Gouty arthritis patients who had multiple nodules in the periarticular and subcutaneous tissue, particularly in toes, fingers, elbows, knees, Achilles tendonsrims and rims of the ears, or had one nodule proven to contain urate crystals by polarized light microscopy were classified as tophaceous gout group. Hypertension was defined as a systolic blood pressure ≥140 mmHg and/or diastolic blood pressure ≥90 mmHg. Additionally, patients receiving antihypertensive medication were also considered hypertensive. DM was defined as a fasting plasma glucose level ≥126 mg/dl. Patients receiving oral hypoglycemic drugs or insulin for DM control were also considered to have DM. Hypercholesterolemia was defined as either the usage of cholesterol-lowering medication or, having a total serum cholesterol ≥250 mg/dl in the absence of cholesterol-lowering medication.

### Echocardiography

According to the guidelines of the American Society of Echocardiograhy [13], all subjects received transthoratic echocardiographic examinations at rest in the left lateral decubitus position using both Philips iE33 ultrasound system (Philips Healthcare, the Netherlands) with a S5-1 transducer and Vivid 7 ultrasound system (GE Vingmed Ultrasound AS, Horten, Norway) with a M4S transducer. The 2D, M-mode and Doppler echocardiographic images were collected from an iE33 ultrasound system to evaluate the structure and function of LV and LA. And the Gray-scales tissue Doppler images of apical four-, three-, and two-chamber views recorded from a Vivid 7 ultrasound system were analyzed to evaluate LA strain, strain rate, and LV strain.

### Assessment of Left Ventricular Volume and Function

Thickness of the interventricular septum (IVS) and posterior wall (PW) in end-diastolic phase, and LV end-diastolic and end-systolic dimensions were determined using the M-mode in the parasternal long-axis view. The LV mass was estimated using the Devereux formula. The LV mass index was obtained based on LV mass/body surface area (BSA). Mitral inflow velocities were evaluated using a 1- to 2-mm sample volume placed at the mitral valve tip by pulse-wave Doppler in the apical four-chamber view. Diastolic peak early (E) and late (A) transmitral flow velocity, and E to A ratio (E/A) were measured. In the apical-four chamber view, the peak early diastolic mitral annular velocity (Em), and peak late diastolic annular velocity (Am) were obtained by pulse-wave tissue Doppler imaging at the septal site of the mitral annulus. The E/Em ratio was calculated for assessment of the LV diastolic dysfunction and was used as an index of LV filling pressure [14]. LV end-diastolic volume (LVEDV), LV end-systolic volume (LVESV), and LV ejection fraction (LVEF) were measured in the apical four-chamber and two-chamber views using the biplane modified Simpson's method. LVEDV and LVESD indexes were calculated as LVEDV/BSA and LVESV/BSA. All echocardiographic parameters were measured using an average of three beats.

### Assessment of Left Atrial Volume and Function

LA volume (LAV) was measured in the apical four-chamber and two-chamber views using the biplane modified Simpson's method. Maximal, precontraction, and minimal LAV were measured just before mitral valve opening, at the beginning of the P wave, and at mitral valve closure, respectively. The following indexes of LA function were calculated: maximal LAV index (LAVi) was calculated as maximal LAV/BSA, precontraction LAVi was calculated as precontraction LAV/BSA, and minimal LAVi was calculated as minimal LAV/BSA. During each cardiac cycle, total LA ejection fraction (LAEF) was defined as (maximal LAV – minimal LAV)/maximal LAV×100%, passive LAEF was defined as (maximal LAV – precontraction LAV)/maximal LAV×100%, and active LAEF was defined as (precontraction LAV – minimal LAV)/precontraction LAV×100%.

### Two Dimentional Speckle Tracking Strain and Strain Rate Analysis

**Figure 1:**
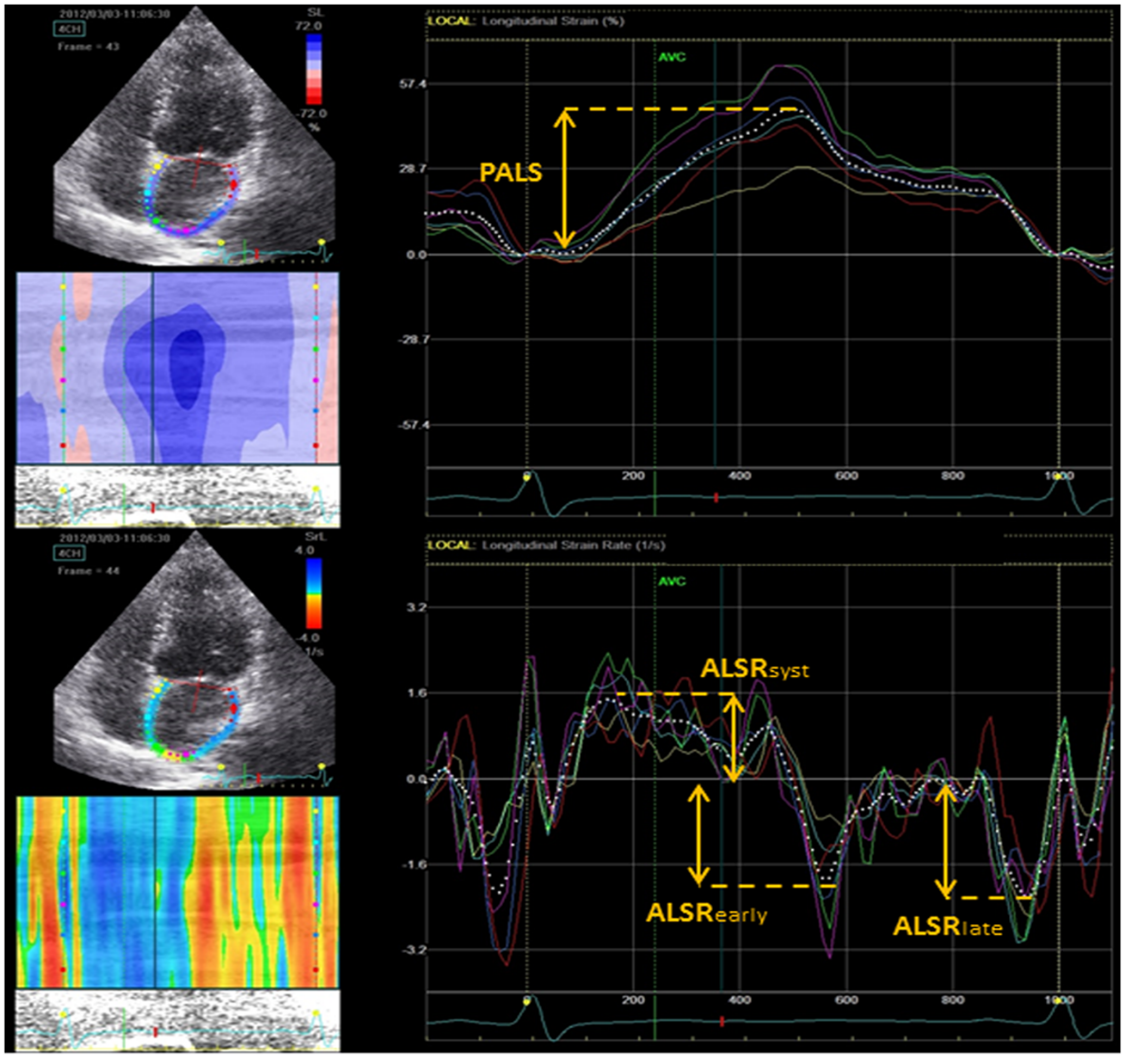
Measurement of left atrial strain (top) and strain rate (bottom). Abbreviations: PALS, Peak atrial longitudinal strain; ALS_syst,_ peak atrial longitudinal strain rate during ventricular systole; ALS_early_, peak atrial longitudinal strain rate during ventricular early diastole; ALS_late_, peak atrial longitudinal strain rate during ventricular late diastole.

For speckle tracking analysis, apical four-, two-, and three-chamber views were gained using echocardiography (Vivid 7, GE). For each view, adequate gray scale images were obtained to outline the endocardial and epicardial border of the LV and the LA. At least three consecutive cardiac cycles were acquired during the patients held breath following complete exhalation with a stable eletrocardiographic recording. Speckle-tracking analysis was performed using EchoPAC software (GE Medical Systems). For each analysis the endocardial border was traced manually, a second line was generated by the software near the epicardium using an automatic default width. We were able to adjust the width to ensure the entire LV or LA myocardial wall was included manually. The region of interest was then divided into six segments and labeled as acceptable or unacceptable based on adequate tracking quality. In poor tracking segments, the endocardial trace line was able to be adjusted until improved tracking quality was achieved. The LV global longitudinal systolic strain (LVε) was the average values of peak longitudinal systolic strain obtained from 17 segments of LV by analysis of the apical four-, two-, and three-chamber views. LA speckle-tracking analysis was performed from the apical four-chamber view to obtain longitudinal LA strain and strain rate. Peak atrial longitudinal strain (PALS) and LA strain rate parameters including ALSR_syst_, ALSR_early_, and ALSR_late_ were assessed as the average of six segmental values in apical four-chamber view demonstrated in Figure 1. ALSR_syst_, ALSR_early_, and ALSR_late_ indicate LA reservoir, conduit and booster pump function.

### Statistics

Statistical analysis was performed using SPSS 18.0 statistical software (SPSS Inc., Chicago, IL). Continuous data are presented as mean ± standard deviation, and dichotomous data as number and percentage. Comparisons of continuous variables between groups were performed using an analysis of variance (ANOVA). Categorical variables were compared using the χ^2^ test. Univariate correlations were determined using Pearson's coefficient for continuous variables. Multiple regression analyses were performed to determine the associations between gout stage with PALS, ALSR_syst_, ALSR_early_, and ALSR_late_. The first multivariate model adjusted for age, UA, creatinine, and LV mass index. The second multivariate model adjusted for E/Em and LVε. Statistical significance was set at P<0.05.

## Results

### General Characteristics

A total of 200 patients were screened for study inclusion. 27 patients were not enrolled because of LV systolic dysfunction (n = 2), hypertrophic cardiomyopathy (n = 2), pacemaker implantation (n = 2), atrial fibrillation (n = 2), and poor echocardiographic images (n = 19). Thus, the final study population consisted of 173 patients. Patients were divided into four groups– stage 0: age-matched controls (n = 35); stage I: asymptomatic hyperuricemia (n = 30), stage II: gouty arthritis without tophi (n = 58); and stage III: tophaceous gout (n = 50). Table 1 lists the clinical characteristics of the study population. There were no significant differences between the four groups with respect to age, gender, and history of DM, hypertension and hypercholesterolemia. There was no significant different urate-lowering therapy between gouty arthritis without tophi group and tophaceous gout group. But high percentage of tophaceous gout patients received steroid therapy to control inflammation. However, UA levels in Stage I, II and III patients were higher compared with control subjects (P<0.001). There were no significant differences in UA levels between patients in stage I, II and III (8.88±1.42 mg/dl vs. 8.11±2.23 mg/dl vs. 8.29±1.84 mg/dl; P = 0.21). Furthermore, stage III patients had significantly worse renal function compared with patients in stage 0, I and II patients (creatinine: 1.63±1.55 mg/dl vs. 0.83±0.20 mg/dl vs. 1.01±0.14 mg/dl vs. 1.09±0.16 mg/dl; P<0.001).

**Table 1:** Clinical characteristics in control subjects and patients with asymptomatic hyperuricemia, gouty arthritis without tophi, and tophaceous gout.

	Stage 0 (n = 35)	Stage I (n = 30)	Stage II (n = 58)	Stage III (n = 50)	P value
Age (years)	53.5±15.64	57.0±16.07	55.8±8.66	61.0±14.15	0.065
Male	26(75%)	27(90%)	52(90%)	45(90%)	0.12
Body surface area (m^2^)	1.69±0.20	1.78±0.17	1.88±0.19*	1.75±0.21‡	<0.001
Uric acid (mg/dl)	4.97±1.05	8.88±1.42*	8.11±2.23*	8.29±1.84*	<0.001
Creatinine (mg/dl)	0.83±0.2	1.01±0.41	1.09±0.16	1.63±1.55†	<0.001
Diabetes mellitus	2(6%)	3(10%)	3(6%)	3(6%)	0.84
Hypertension	8(23%)	8(27%)	9(16%)	13(26%)	0.515
Hypercholesterolemia	9(26%)	8(27%)	21(37%)	15(30%)	0.689
Drugs					
Allopurinol			43(75%)	44(88%)	0.07
Benzbromazone			10(18%)	8(16%)	0.863
Sulfinpyrazone			11(19%)	8(16%)	0.678
Colchicine			38(66%)	39(78%)	0.153
Prednisolone			7(12%)	15(30%)	0.021

Data are expressed as mean± standard deviation or number (percentage). Stage 0: age-match control, Stage I: asymptomatic hyperuricemia, Stage II: gouty arthritis without tophi, Stage III: tophaceous gout.

*P<0.05 versus stage 0.

† P<0.05 versus stage 0, I and II.

‡ P<0.05 versus stage II.

### Left Ventricular Echocardiographic Parameters

The LV echocardiographic parameters are summarized in Table 2. Stage III patients had significantly thicker IVS (10.86±1.73 vs. 9.37±1.61 mm; P = 0.001) and PW (11.27±1.7 vs. 9.89±1.57 mm; P = 0.003) compared with controls. The LV mass index in stage III patients was significantly higher than in stage 0, I and II patients (117.71±31.00 vs. 91.14±27.03 vs. 100.56±24.10 vs. 98.11±25.07 g/m^2^; P<0.001). There were no significant differences between the four groups with respect to LV end-diastolic dimensions, end-systolic dimensions, LVEDV, and LVESV. Additionally, there were no significant differences in LV systolic function measured by biplane modified Simpson's method between the four groups. The E, A, E/A ratio, and Am were not significantly different between the four groups. The stage III patients had a significantly lower Em compared with controls (6.50±2.04 vs. 8.79±3.05; P<0.001). Lastly, the Em/Am ratio was lower in stage III patients compared with controls (0.75±0.31 vs. 1.14±0.57; P<0.001) and the E/Em ratio was higher in the stage III patients compared with controls (10.50±3.18 vs. 8.58±2.07; P = 0.008). The absolute value of the LVε in stage II and III is lower than in stage 0 and I (P = 0.002).

**Table 2:** Left ventricular echocardiographic characteristics in control subjects and patients with asymptomatic hyperuricemia, gouty arthritis without tophi, and tophaceous gout.

	Stage 0	Stage I	Stage II	Stage III	P value
IVS(mm)	9.37±1.61	10.27±1.86	10.17±1.54	10.86±1.73*	0.001
LVEDd(mm)	45.81±6.11	47.16±4.48	48.15±5.07	48.42±6.07	0.138
PW(mm)	9.89±1.57	10.65±1.71	10.64±1.55	11.27±1.71*	0.003
LVESd(mm)	25.64±4.77	27.32±3.77	27.02±4.30	27.16±4.52	0.346
LV mass(g/m)	154.11±48.92	177.80±41.78	184.00±47.84	205.38±52.32*	<0.001
LV mass index(g/m^2^)	91.14±27.03	100.56±24.10	98.11±25.07	117.71±31.00†	<0.001
LVEDV(ml)	66.97±20.20	68.80±19.81	76.12±21.15	76.92±18.34	0.053
LVEDV index(ml/m^2^)	39.56±10.34	38.51±9.88	40.50±10.96	44.35±11.05	0.067
LVESV(ml)	18.54±7.29	19.67±9.26	20.97±7.86	22.24±8.00	0.185
LVESV index(ml/m^2^)	10.96±3.92	10.93±4.85	11.22±4.50	12.87±5.08	0.145
LVEF(%)	72.57±5.43	72.50±6.98	72.60±5.81	71.20±6.09	0.618
E(m/s)	0.71±0.16	0.65±0.15	0.70±0.14	0.65±0.17	0.173
A(m/s)	0.72±0.17	0.75±0.23	0.70±0.18	0.79±0.21	0.083
E/A	1.05±0.39	0.93±0.31	1.06±0.32	0.89±0.36	0.06
Em(cm/s)	8.79±3.05	7.95±2.15	7.50±1.92	6.50±2.04*	<0.001
Am (cm/s)	8.25±1.55	9.02±2.14	8.96±1.74	9.02±2.04	0.219
Em/Am	1.14±0.57	0.95±0.41	0.88±0.32*	0.75±0.31*	<0.001
E/Em	8.58±2.07	8.79±3.31	9.70±2.64	10.50±3.18*	0.008
LVε(%)	−21.79±2.27	−21.99±2.18	−20.28±2.7* ‡	−20.2±3.06* ‡	0.002

Data are expressed as mean±standard deviation or number (percentage). Stage 0: age-match control, Stage I: asymptomatic hyperuricemia, Stage II: gouty arthritis without tophi, Stage III: tophaceous gout. LV, left ventricle; IVS, interventricular septum; LVEDd, LV end-diastolic diameter; PW, posterior wall thickness; LVESd, LV end-systolic diameter; LVEDV, LV end-diastolic volume; LVESV, LV end-systolic volume; LVEF, LV ejection fraction; E, early diastolic peak transmitral flow velocity; A, late diastolic peak transmitral flow velocity; Em, peak early diastolic mitral annular velocity; Am, peak late diastolic annular velocity; LVε, LV longitudinal strain.

*P<0.05 versus Stage 0.

† P<0.05 versus Stage 0, Stage I and Stage II.

‡ P<0.05 versus Stage I.

### Left Atrial Volume and Function

The echocardiographic parameters in terms of LA volume and function are listed in Table 3. Maximal, precontraction, and minimal LAVi were progressively enlarged from the control group to those patients in the stage III group. Additionally, maximal, precontraction, and minimal LAVi in stage III patients was significantly larger than in controls (29.60±9.89 vs. 20.07±4.76, 19.10±8.46 vs. 11.50±3.37, 9.58±4.23 vs. 6.13±2.15; all P<0.001). LA function, as evaluated by total, passive, and active LAEF was not different among the four groups. Conversely, the value of PALS, ALSR_syst_, ALSR_early_, and ALSR_late_ decreased progressively from the control group to those patients in stage III. And the value of PALS, ALSR_syst_, ALSR_early_, and ALSR_late_ in stage III patients was significantly lower compared with patients in the control group (38.97±10.85 vs. 45.61±13.21, 1.73±0.48 vs. 2.05±0.55, −1.44±0.53 vs. −2.07±0.84, −2.07±0.7 vs. −2.66±0.91; all P<0.05).

**Table 3:** Left atrial echocardiographic characteristics in control subjects and patients with asymptomatic hyperuricemia, gouty arthritis without tophi, and tophaceous gout.

	Stage 0	Stage I	Stage II	Stage III	P value
**LA volume**					
Maximal LAVi (ml/m^2^)	20.07±4.76	20.24±5.02	24.39±7.94	29.60±9.89†	<0.001
Precontraction LAVi (ml/m^2^)	11.50±3.37	12.72±4.21	15.13±5.80	19.10±8.46†	<0.001
Minimal LAVi (ml/m^2^)	6.13±2.15	6.37±2.13	8.17±4.12	9.58±4.23‡	<0.001
**LA function**					
Total LAEF(%)	69.68±6.51	68.91±6.22	67.21±8.92	67.91±8.80	0.503
Passive LAEF(%)	42.67±9.88	38.00±9.28	38.31±9.71	37.02±10.97	0.074
Active LAEF(%)	47.16±6.89	49.49±9.64	47.01±10.45	48.63±11.89	0.654
PALS (%)	45.61±13.21	43.32±7.81	44.45±12.03	38.97±10.85*	0.03
ALS_syst_ (1/s)	2.05±0.55	2.08±0.38	1.96±0.49	1.73±0.48‡	0.004
ALS_early_ (1/s)	−2.07±0.84	−1.88±0.71	−1.80±0.47	−1.44±0.53†	<0.001
ALS_late_ (1/s)	−2.66±0.91	−2.58±0.69	−2.59±0.7	−2.07±0.7†	0.001

Data are expressed as mean±standard deviation or number (percentage). Stage 0: age-match control, Stage I: asymptomatic hyperuricemia, Stage II: gouty arthritis without tophi, Stage III: tophaceous gout. LA, left atrium; LAVi, LA volume index; LAEF, LA ejection fraction; PALS, Peak atrial longitudinal strain; ALS-syst, peak atrial longitudinal strain rate during left ventricular systole; ALS-early, peak atrial longitudinal strain rate during ventricular early diastole; ALS-late, peak atrial longitudinal strain rate during ventricular late diastole.

*P<0.05 versus Stage 0.

† P<0.05 versus Stage 0, Stage I and Stage II.

‡ P<0.05 versus Stage 0 and Stage I.

### Regression Analysis

**Figure 2:**
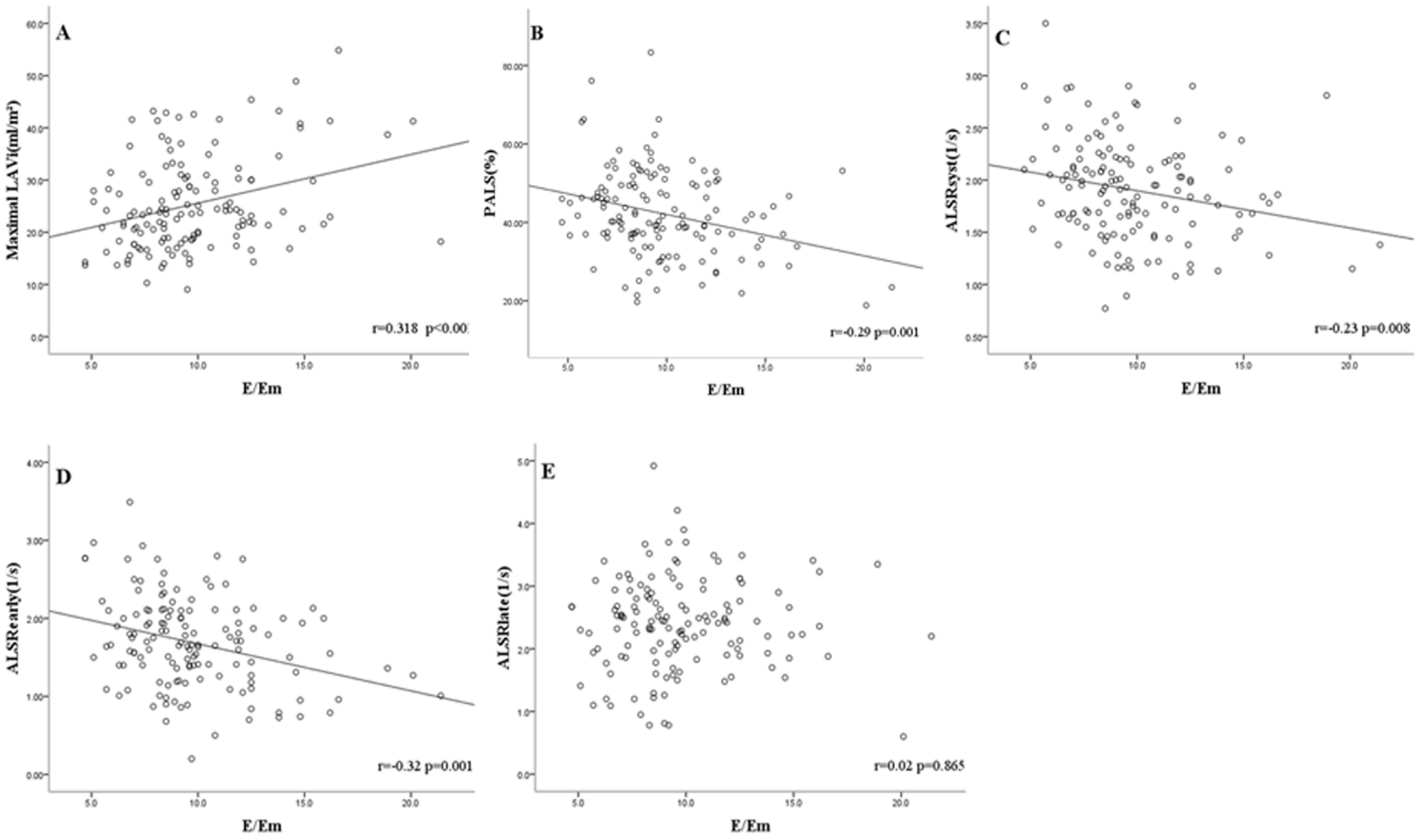
Linear regression analysis and Pearson correlation coefficients between E/Em and maximal LAVi (A),PALS (B), ALSR_syst_ (C), ALSR_early_ (D) and ALSR_late_ (E) in patients diagnosed with gout (n = 138). Abbreviations: LAVi, left atrial volume index; PALS, Peak atrial longitudinal strain; ALS_syst_, peak atrial longitudinal strain rate during ventricular systole; ALS_early_, peak atrial longitudinal strain rate during ventricular early diastole; ALS_late_, peak atrial longitudinal strain rate during ventricular late diastole.

Maximal LAVi, PALS, ALSR_syst_, and ALSR_early_ were significantly correlated with E/Em (r = 0.318, −0.29, −0.23 and −0.32, respectively; all P<0.01) in gout patients including asymptomatic hyperuricemia, gouty arthritis without tophi and tophaceous gout. Conversely, ALSR_late_ was not correlated with E/Em (r = 0.02, P = 0.865) (Figure 2). The multivariate analysis (Table 4) also showed that the stage of gout had a negative impact on ALSR_late_ in each study group.

**Table 4:** Multivariate regression analysis of the association between left atrial functional parameters and gout stage between study groups (n = 173).

	Unadjusted*		Model 1†		Model 2‡	
Variable	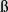	P	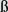	P	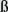	P
PALS (%)						
Gout stage	−0.184	0.016	−0.081	0.361	−0.1	0.913
ALS-syst (1/s)						
Gout stage	−0.24	0.001	−0.172	0.054	−0.116	0.195
ALS-early (1/s)						
Gout stage	−0.337	<0.001	−0.112	0.113	−0.09	0.216
ALS-late (1/s)						
Gout stage	−0.256	0.001	−0.21	0.019	−0.175	0.048

PALS, Peak atrial longitudinal strain; ALS-syst, peak atrial longitudinal strain rate during ventricular systole; ALS-early, peak atrial longitudinal strain rate during ventricular early diastole; ALS-late, peak atrial longitudinal strain rate during ventricular late diastole. Absolute values of ALS-early and ALS-late were used in multivariate regressions.

*Including gout stage.

† Adjusted for age, uric acid, creatinine and left ventricular (LV) mass index.

‡ Adjusted as model 1, pluse ratio of the transmitral and myocardial peak early diastolic velocities (E/Em), LV global longitudinal systolic strain (LVε).

In 20 randomly selected subjects, the interobserver and intraobserver variability in E/Em, maximal LAVi and ALSR_late_ measurements were assessed by the coefficient of variation, where differences between measurements were expressed as the ratio of the standard deviation to the mean. Inter-observer variability was assessed using two independent observers and intra-observer variability using one observer twice within a two-week period. The inter-observer variability of E/Em was 3.3%, maximal LAV was 2.2% and ALSR_late_ was 6%. The intra-observer variability of E/Em was 1.8%, maximal LAV was 1.5% and ALSR_late_ was 5%.

## Discussion

The major findings of this study was that severity of gout caused LV diastolic dysfunction, subclinical systolic dysfunction and LA reservoir, conduit, booster pump dysfunction and LA volume enlargement.

### Gout, not only hyperuricemia, linked to LV functional remodeling

Gout is a metabolic disorder characterized by hyperuricemia and is a type of chronic inflammatory arthritis induced not only by xanthine oxidase-mediated oxidative stress, but also by the deposition of monosodium urate (MSU) crystals in synovial fluid and other tissue [1]. High UA levels has been demonstrated to be associated with increased LV mass, end-diastolic LV dimensions, and IVS thickness compared with patients with low UA levels [15]. A possible mechanism to explain this observed relationship was the accumulation of reactive oxygen species due to the up-regulation of xanthine oxidase with hyperuricemia. However, in patients with gout, MSU crystals also mediate local and systemic inflammatory processes which can induce many inflammatory cytokines, including tumor necrosis factor α (TNF-α), interleukin-1β (IL-1β), interleukin-6, and interleukin-8 [1]. Gout flares occur as a result of these MSU crystal deposits in the joints being suddenly released and setting off an inflammatory cascade that manifests as an acute gouty arthritis attack [16]–[17], and MSU crystals cause persistent low-grade inflammation during the intercritical period [18]. In the previous study, TNF-α and IL-1β have been implicated in the pathogenesis of myocardial dysfunction in chronic heart failure, and are suspected to mediate LV remodeling [19]. Clinically, a positive association between gout and cardiovascular events has been observed [20]. A recent study also showed gout, not hyperuricemia, was linked to a high risk of cardiovascular diseases [21]. Our study is to evaluate cardiac function in different stages of gout. Currently, UA levels were not significantly different between the asymptomatic hyperuricemia, gouty arthritis without tophi, and tophaceous gout groups (Table 1). Thus serum UA levels do not reflect the severity of gout and inflammation. The creatinine levels of the tophaceous gout group were significantly higher than those of the control, asymptomatic hyperuricemia and gouty arthritis without tophi groups (Table 1). These findings suggest that tophaceous gout patients suffered from a longer period of inflammation mediated by MSU crystals and had impaired renal function.

As the LV undergo remodeling, the LV filling pressure increases. The E/Em reflects the LV filling pressure and is a parameter of LV diastolic function. The higher E/Em, the poor LV diastolic function [22]. LV myocardial deformation assessed by strain (LVε) is the result of a complex interaction between the intrinsic contractile force and the extrinsic loading condition which can reflect the subclinical LV systolic functional remodeling [23]. In our study, tophaceous gout patients had worse LV diastolic function (higher E/Em) compared with controls. LV diastolic dysfunction is frequently associated with LV hypertrophy. In our study, we also found that the LV mass index in the tophaceous gout group was significantly higher than that of the control, asymptomatic hyperuricemia and gouty arthritis without tophi groups. The LVε in gouty arthritis without tophi and tophaceous gout groups were significantly lower compared with control and asymptomatic hyperuricemia groupswhile the LVEF was similar in control, asymptomatic hyperuricemia, gouty arthritis without tophi and tophaceous gout groups. These results suggest that in patients with gout, LV remodeling including LV diastolic dysfunction and subclinical LV systolic dysfunction could be associated with the ongoing inflammation mediated by MSU crystals rather than isolated hyperuricemia.

### LA dysfunction and increased LA volume in gout patients

The LA acts as a reservoir during LV systole, as a conduit in LV early diastole, and as a booster pump in the LV during late diastole. Reduced LA reservoir and booster pump functions can reflect the progressive deterioration of LV diastolic dysfunction [24]. In the current study, total LAEF, passive LAEF, and active LAEF were not different between study groups, however the three phases of LA function including reservoir, conduit, and booster pump were significantly reduced in patients with tophaceous gout. LA enlargement is the result of pressure and volume overload and can be an index of LA remodeling [25]. In this study we demonstrated that maximal LAVi, pre-contraction LAVi and minimal LAVi increased from the control group to tophaceous gout group progressively. Gout, not solely hyeruricemia, is also associated with increased LA maximal volume and decreased LA reservoir, conduit, and booster pump functions.

In this study, maximal LAVi, PALS, ALSR_syst_, and ALSR_early_ were significantly correlated with E/Em, except ALSR_late_ (Figure 2). This suggests that gout impairs LV compliance which increased LV filling pressure. During LV diastolic phase, the LA directly exposed to high LV pressure which decreased PALS, ALSR_syst_, ALSR_early_ and increased maximal LAVi through the open mitral valve. Using multivariate analysis, gout stage was an independent of decreased LA booster pump function (ALSR_late)_. These results suggest LA booster pump is not only modulated by impaired LV diastolic function but also modulated by ongoing inflammation mediated by MSU crystals in patients with gout. As the progressive severity and inflammation of gout, the LA booster pump function decreased. Reduced LA booster pump function has been demonstrated to predict new-onset atrial fibrillation [26], and has emerged as an independent correlate of heart failure symptoms [27]. Gout patients with reduced LA booster pump function may have high risk of adverse cardiovascular events.

### Study Limitations

There are several limitations in the present study. First, it was a cross-sectional study, further follow-up cohort studies are necessary to demonstrate a reduced LA booster pump is a predictive factor of adverse cardiac events in patients with gout. Secondly, the biological link between gout and organic heart remodeling remains unclear, our current hypotheses and results require further clarification through additional studies.

## Conclusions

Severity of gout was significantly associated with the deterioration of LV diastolic dysfunction, LV subclinical systolic dysfunction, enlarged LA volume, and impaired LA function including reservoir, conduit, and booster pump functions.
